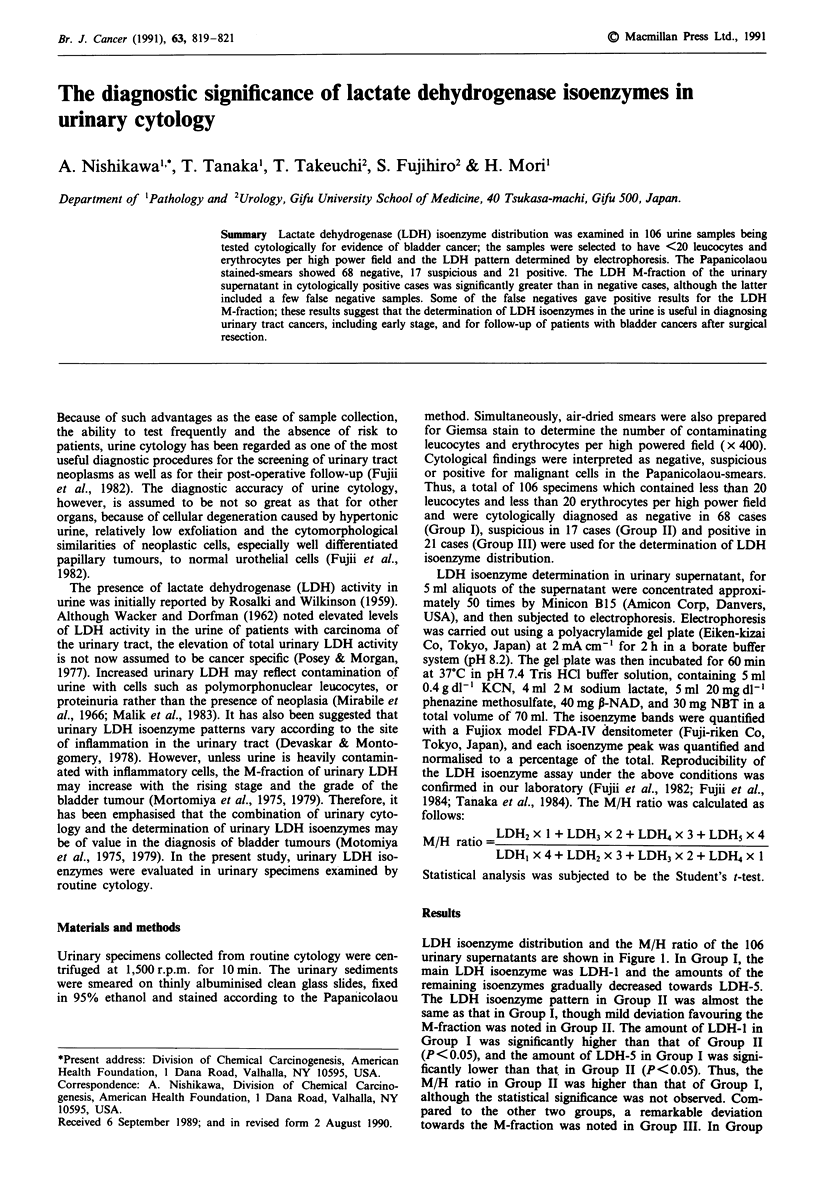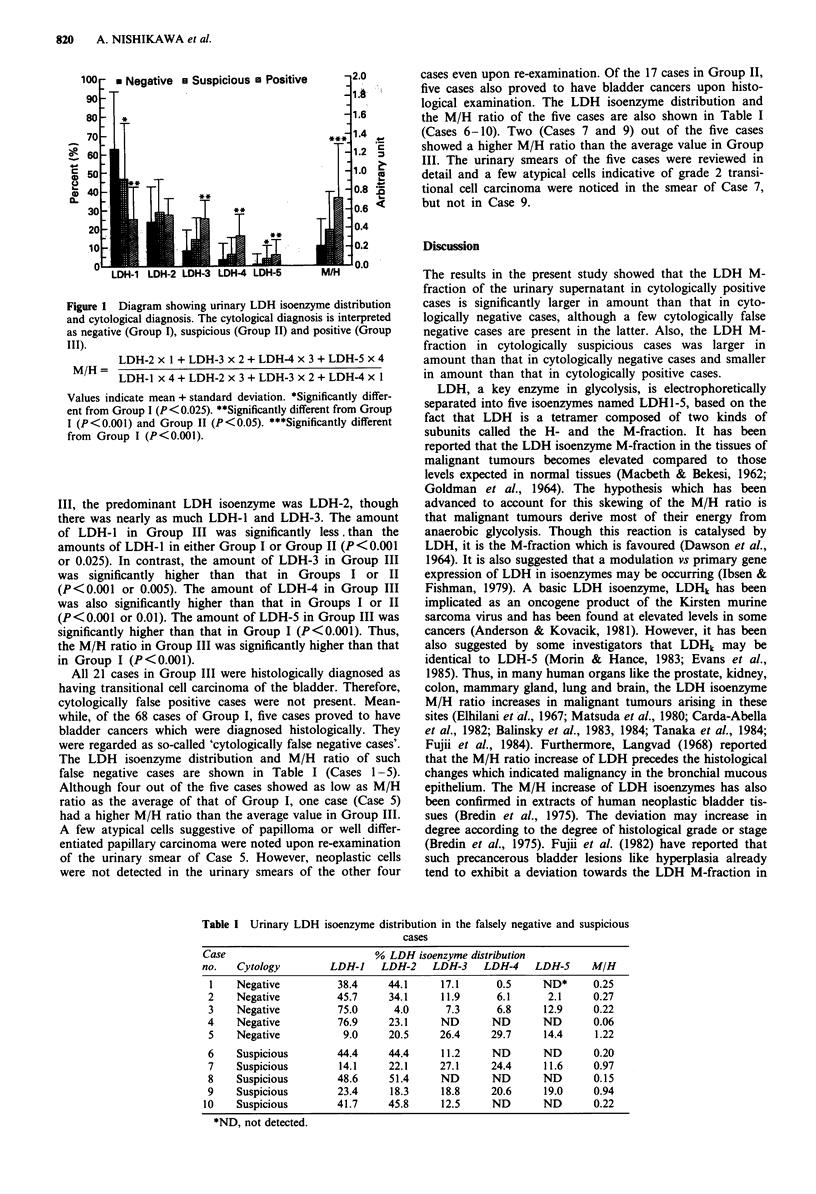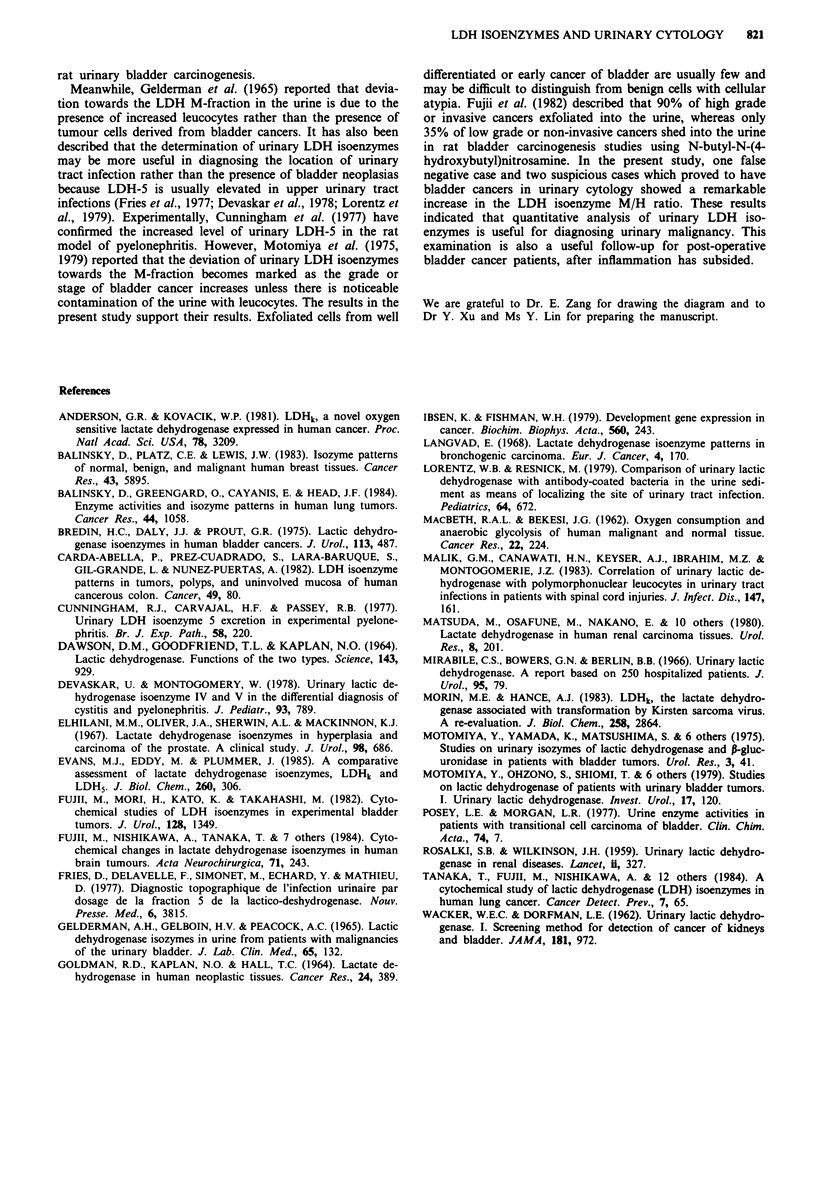# The diagnostic significance of lactate dehydrogenase isoenzymes in urinary cytology.

**DOI:** 10.1038/bjc.1991.181

**Published:** 1991-05

**Authors:** A. Nishikawa, T. Tanaka, T. Takeuchi, S. Fujihiro, H. Mori

**Affiliations:** Department of Pathology, Gifu University School of Medicine, Japan.

## Abstract

Lactate dehydrogenase (LDH) isoenzyme distribution was examined in 106 urine samples being tested cytologically for evidence of bladder cancer; the samples were selected to have less than 20 leucocytes and erythrocytes per high power field and the LDH pattern determined by electrophoresis. The Papanicolaou stained-smears showed 68 negative, 17 suspicious and 21 positive. The LDH M-fraction of the urinary supernatant in cytologically positive cases was significantly greater than in negative cases, although the latter included a few false negative samples. Some of the false negatives gave positive results for the LDH M-fraction; these results suggest that the determination of LDH isoenzymes in the urine is useful in diagnosing urinary tract cancers, including early stage, and for follow-up of patients with bladder cancers after surgical resection.


© Macmillan Press Ltd., 1991

### The diagnostic significance of lactate dehydrogenase isoenzymes in urinary cytology

A. Nishikawa[1,*], T. Tanaka[1], T. Takeuchi[2], S. Fujihiro[2] & H. Mori[1]

*Department of [1]Pathology and [2]Urology, Gifu University School of Medicine, 40 Tsukasa-machi, Gifu 500, Japan.*

**Summary** Lactate dehydrogenase (LDH) isoenzyme distribution was examined in 106 urine samples being tested cytologically for evidence of bladder cancer; the samples were selected to have <20 leucocytes and erythrocytes per high power field and the LDH pattern determined by electrophoresis. The Papanicolaou stained-smears showed 68 negative, 17 suspicious and 21 positive. The LDH M-fraction of the urinary supernatant in cytologically positive cases was significantly greater than in negative cases, although the latter included a few false negative samples. Some of the false negatives gave positive results for the LDH M-fraction; these results suggest that the determination of LDH isoenzymes in the urine is useful in diagnosing urinary tract cancers, including early stage, and for follow-up of patients with bladder cancers after surgical resection.

Because of such advantages as the ease of sample collection, the ability to test frequently and the absence of risk to patients, urine cytology has been regarded as one of the most useful diagnostic procedures for the screening of urinary tract neoplasms as well as for their post-operative follow-up (Fujii *et al.*, 1982). The diagnostic accuracy of urine cytology, however, is assumed to be not so great as that for other organs, because of cellular degeneration caused by hypertonic urine, relatively low exfoliation and the cytomorphological similarities of neoplastic cells, especially well differentiated papillary tumours, to normal urothelial cells (Fujii *et al.*, 1982).

The presence of lactate dehydrogenase (LDH) activity in urine was initially reported by Rosalki and Wilkinson (1959). Although Wacker and Dorfman (1962) noted elevated levels of LDH activity in the urine of patients with carcinoma of the urinary tract, the elevation of total urinary LDH activity is not now assumed to be cancer specific (Posey & Morgan, 1977). Increased urinary LDH may reflect contamination of urine with cells such as polymorphonuclear leucocytes, or proteinuria rather than the presence of neoplasia (Mirabile *et al.*, 1966; Malik *et al.*, 1983). It has also been suggested that urinary LDH isoenzyme patterns vary according to the site of inflammation in the urinary tract (Devaskar & Montogomery, 1978). However, unless urine is heavily contaminated with inflammatory cells, the M-fraction of urinary LDH may increase with the rising stage and the grade of the bladder tumour (Mortomiya *et al.*, 1975, 1979). Therefore, it has been emphasised that the combination of urinary cytology and the determination of urinary LDH isoenzymes may be of value in the diagnosis of bladder tumours (Motomiya *et al.*, 1975, 1979). In the present study, urinary LDH isoenzymes were evaluated in urinary specimens examined by routine cytology.

### Materials and methods

Urinary specimens collected from routine cytology were centrifuged at 1,500 r.p.m. for 10 min. The urinary sediments were smeared on thinly albuminised clean glass slides, fixed in 95% ethanol and stained according to the Papanicolaou method. Simultaneously, air-dried smears were also prepared for Giemsa stain to determine the number of contaminating leucocytes and erythrocytes per high powered field (× 400). Cytological findings were interpreted as negative, suspicious or positive for malignant cells in the Papanicolaou-smears. Thus, a total of 106 specimens which contained less than 20 leucocytes and less than 20 erythrocytes per high power field and were cytologically diagnosed as negative in 68 cases (Group I), suspicious in 17 cases (Group II) and positive in 21 cases (Group III) were used for the determination of LDH isoenzyme distribution.

LDH isoenzyme determination in urinary supernatant, for 5 ml aliquots of the supernatant were concentrated approximately 50 times by Minicon B15 (Amicon Corp, Danvers, USA), and then subjected to electrophoresis. Electrophoresis was carried out using a polyacrylamide gel plate (Eiken-kizai Co, Tokyo, Japan) at 2 mA $cm^{-1}$ for 2 h in a borate buffer system (pH 8.2). The gel plate was then incubated for 60 min at 37°C in pH 7.4 Tris HCl buffer solution, containing 5 ml 0.4 g $dl^{-1}$ KCN, 4 ml 2 M sodium lactate, 5 ml 20 mg $dl^{-1}$ phenazine methosulfate, 40 mg β-NAD, and 30 mg NBT in a total volume of 70 ml. The isoenzyme bands were quantified with a Fujiox model FDA-IV densitometer (Fuji-riken Co, Tokyo, Japan), and each isoenzyme peak was quantified and normalised to a percentage of the total. Reproducibility of the LDH isoenzyme assay under the above conditions was confirmed in our laboratory (Fujii *et al.*, 1982; Fujii *et al.*, 1984; Tanaka *et al.*, 1984). The M/H ratio was calculated as follows:

$$\text{M/H ratio} = \frac{LDH_2 \times 1 + LDH_3 \times 2 + LDH_4 \times 3 + LDH_5 \times 4}{LDH_1 \times 4 + LDH_2 \times 3 + LDH_3 \times 2 + LDH_4 \times 1}$$

Statistical analysis was subjected to be the Student's *t*-test.

### Results

LDH isoenzyme distribution and the M/H ratio of the 106 urinary supernatants are shown in Figure 1. In Group I, the main LDH isoenzyme was LDH-1 and the amounts of the remaining isoenzymes gradually decreased towards LDH-5. The LDH isoenzyme pattern in Group II was almost the same as that in Group I, though mild deviation favouring the M-fraction was noted in Group II. The amount of LDH-1 in Group I was significantly higher than that of Group II ($P<0.05$), and the amount of LDH-5 in Group I was significantly lower than that in Group II ($P<0.05$). Thus, the M/H ratio in Group II was higher than that of Group I, although the statistical significance was not observed. Compared to the other two groups, a remarkable deviation towards the M-fraction was noted in Group III. In Group

*Present address: Division of Chemical Carcinogenesis, American Health Foundation, 1 Dana Road, Valhalla, NY 10595, USA.
Correspondence: A. Nishikawa, Division of Chemical Carcinogenesis, American Health Foundation, 1 Dana Road, Valhalla, NY 10595, USA.
Received 6 September 1989; and in revised form 2 August 1990.

**Figure 1** Diagram showing urinary LDH isoenzyme distribution and cytological diagnosis. The cytological diagnosis is interpreted as negative (Group I), suspicious (Group II) and positive (Group III).

$$M/H = \frac{LDH\text{-}2 \times 1 + LDH\text{-}3 \times 2 + LDH\text{-}4 \times 3 + LDH\text{-}5 \times 4}{LDH\text{-}1 \times 4 + LDH\text{-}2 \times 3 + LDH\text{-}3 \times 2 + LDH\text{-}4 \times 1}$$

Values indicate mean + standard deviation. *Significantly different from Group I ($P<0.025$). **Significantly different from Group I ($P<0.001$) and Group II ($P<0.05$). ***Significantly different from Group I ($P<0.001$).

III, the predominant LDH isoenzyme was LDH-2, though there was nearly as much LDH-1 and LDH-3. The amount of LDH-1 in Group III was significantly less than the amounts of LDH-1 in either Group I or Group II ($P<0.001$ or 0.025). In contrast, the amount of LDH-3 in Group III was significantly higher than that in Groups I or II ($P<0.001$ or 0.005). The amount of LDH-4 in Group III was also significantly higher than that in Groups I or II ($P<0.001$ or 0.01). The amount of LDH-5 in Group III was significantly higher than that in Group I ($P<0.001$). Thus, the M/H ratio in Group III was significantly higher than that in Group I ($P<0.001$).

All 21 cases in Group III were histologically diagnosed as having transitional cell carcinoma of the bladder. Therefore, cytologically false positive cases were not present. Meanwhile, of the 68 cases of Group I, five cases proved to have bladder cancers which were diagnosed histologically. They were regarded as so-called 'cytologically false negative cases'. The LDH isoenzyme distribution and M/H ratio of such false negative cases are shown in Table I (Cases 1–5). Although four out of the five cases showed as low as M/H ratio as the average of that of Group I, one case (Case 5) had a higher M/H ratio than the average value in Group III. A few atypical cells suggestive of papilloma or well differentiated papillary carcinoma were noted upon re-examination of the urinary smear of Case 5. However, neoplastic cells were not detected in the urinary smears of the other four cases even upon re-examination. Of the 17 cases in Group II, five cases also proved to have bladder cancers upon histological examination. The LDH isoenzyme distribution and the M/H ratio of the five cases are also shown in Table I (Cases 6–10). Two (Cases 7 and 9) out of the five cases showed a higher M/H ratio than the average value in Group III. The urinary smears of the five cases were reviewed in detail and a few atypical cells indicative of grade 2 transitional cell carcinoma were noticed in the smear of Case 7, but not in Case 9.

### Discussion

The results in the present study showed that the LDH M-fraction of the urinary supernatant in cytologically positive cases is significantly larger in amount than that in cytologically negative cases, although a few cytologically false negative cases are present in the latter. Also, the LDH M-fraction in cytologically suspicious cases was larger in amount than that in cytologically negative cases and smaller in amount than that in cytologically positive cases.

LDH, a key enzyme in glycolysis, is electrophoretically separated into five isoenzymes named LDH1-5, based on the fact that LDH is a tetramer composed of two kinds of subunits called the H- and the M-fraction. It has been reported that the LDH isoenzyme M-fraction in the tissues of malignant tumours becomes elevated compared to those levels expected in normal tissues (Macbeth & Bekesi, 1962; Goldman *et al.*, 1964). The hypothesis which has been advanced to account for this skewing of the M/H ratio is that malignant tumours derive most of their energy from anaerobic glycolysis. Though this reaction is catalysed by LDH, it is the M-fraction which is favoured (Dawson *et al.*, 1964). It is also suggested that a modulation *vs* primary gene expression of LDH in isoenzymes may be occurring (Ibsen & Fishman, 1979). A basic LDH isoenzyme, $LDH_k$ has been implicated as an oncogene product of the Kirsten murine sarcoma virus and has been found at elevated levels in some cancers (Anderson & Kovacik, 1981). However, it has been also suggested by some investigators that $LDH_k$ may be identical to LDH-5 (Morin & Hance, 1983; Evans *et al.*, 1985). Thus, in many human organs like the prostate, kidney, colon, mammary gland, lung and brain, the LDH isoenzyme M/H ratio increases in malignant tumours arising in these sites (Elhilani *et al.*, 1967; Matsuda *et al.*, 1980; Carda-Abella *et al.*, 1982; Balinsky *et al.*, 1983, 1984; Tanaka *et al.*, 1984; Fujii *et al.*, 1984). Furthermore, Langvad (1968) reported that the M/H ratio increase of LDH precedes the histological changes which indicated malignancy in the bronchial mucous epithelium. The M/H increase of LDH isoenzymes has also been confirmed in extracts of human neoplastic bladder tissues (Bredin *et al.*, 1975). The deviation may increase in degree according to the degree of histological grade or stage (Bredin *et al.*, 1975). Fujii *et al.* (1982) have reported that such precancerous bladder lesions like hyperplasia already tend to exhibit a deviation towards the LDH M-fraction in

**Table I** Urinary LDH isoenzyme distribution in the falsely negative and suspicious cases

| *Case no.* | *Cytology* | *% LDH isoenzyme distribution* *LDH-1* | *LDH-2* | *LDH-3* | *LDH-4* | *LDH-5* | *M/H* |
|---|---|---|---|---|---|---|---|
| 1 | Negative | 38.4 | 44.1 | 17.1 | 0.5 | ND* | 0.25 |
| 2 | Negative | 45.7 | 34.1 | 11.9 | 6.1 | 2.1 | 0.27 |
| 3 | Negative | 75.0 | 4.0 | 7.3 | 6.8 | 12.9 | 0.22 |
| 4 | Negative | 76.9 | 23.1 | ND | ND | ND | 0.06 |
| 5 | Negative | 9.0 | 20.5 | 26.4 | 29.7 | 14.4 | 1.22 |
| 6 | Suspicious | 44.4 | 44.4 | 11.2 | ND | ND | 0.20 |
| 7 | Suspicious | 14.1 | 22.1 | 27.1 | 24.4 | 11.6 | 0.97 |
| 8 | Suspicious | 48.6 | 51.4 | ND | ND | ND | 0.15 |
| 9 | Suspicious | 23.4 | 18.3 | 18.8 | 20.6 | 19.0 | 0.94 |
| 10 | Suspicious | 41.7 | 45.8 | 12.5 | ND | ND | 0.22 |

*ND, not detected.

rat urinary bladder carcinogenesis.

Meanwhile, Gelderman *et al.* (1965) reported that deviation towards the LDH M-fraction in the urine is due to the presence of increased leucocytes rather than the presence of tumour cells derived from bladder cancers. It has also been described that the determination of urinary LDH isoenzymes may be more useful in diagnosing the location of urinary tract infection rather than the presence of bladder neoplasias because LDH-5 is usually elevated in upper urinary tract infections (Fries *et al.*, 1977; Devaskar *et al.*, 1978; Lorentz *et al.*, 1979). Experimentally, Cunningham *et al.* (1977) have confirmed the increased level of urinary LDH-5 in the rat model of pyelonephritis. However, Motomiya *et al.* (1975, 1979) reported that the deviation of urinary LDH isoenzymes towards the M-fraction becomes marked as the grade or stage of bladder cancer increases unless there is noticeable contamination of the urine with leucocytes. The results in the present study support their results. Exfoliated cells from well differentiated or early cancer of bladder are usually few and may be difficult to distinguish from benign cells with cellular atypia. Fujii *et al.* (1982) described that 90% of high grade or invasive cancers exfoliated into the urine, whereas only 35% of low grade or non-invasive cancers shed into the urine in rat bladder carcinogenesis studies using N-butyl-N-(4-hydroxybutyl)nitrosamine. In the present study, one false negative case and two suspicious cases which proved to have bladder cancers in urinary cytology showed a remarkable increase in the LDH isoenzyme M/H ratio. These results indicated that quantitative analysis of urinary LDH isoenzymes is useful for diagnosing urinary malignancy. This examination is also a useful follow-up for post-operative bladder cancer patients, after inflammation has subsided.

We are grateful to Dr. E. Zang for drawing the diagram and to Dr Y. Xu and Ms Y. Lin for preparing the manuscript.

### References

ANDERSON, G.R. & KOVACIK, W.P. (1981). $LDH_k$, a novel oxygen sensitive lactate dehydrogenase expressed in human cancer. *Proc. Natl Acad. Sci. USA*, **78**, 3209.

BALINSKY, D., PLATZ, C.E. & LEWIS, J.W. (1983). Isozyme patterns of normal, benign, and malignant human breast tissues. *Cancer Res.*, **43**, 5895.

BALINSKY, D., GREENGARD, O., CAYANIS, E. & HEAD, J.F. (1984). Enzyme activities and isozyme patterns in human lung tumors. *Cancer Res.*, **44**, 1058.

BREDIN, H.C., DALY, J.J. & PROUT, G.R. (1975). Lactic dehydrogenase isoenzymes in human bladder cancers. *J. Urol.*, **113**, 487.

CARDA-ABELLA, P., PREZ-CUADRADO, S., LARA-BARUQUE, S., GIL-GRANDE, L. & NUNEZ-PUERTAS, A. (1982). LDH isoenzyme patterns in tumors, polyps, and uninvolved mucosa of human cancerous colon. *Cancer*, **49**, 80.

CUNNINGHAM, R.J., CARVAJAL, H.F. & PASSEY, R.B. (1977). Urinary LDH isoenzyme 5 excretion in experimental pyelonephritis. *Br. J. Exp. Path.*, **58**, 220.

DAWSON, D.M., GOODFRIEND, T.L. & KAPLAN, N.O. (1964). Lactic dehydrogenase. Functions of the two types. *Science*, **143**, 929.

DEVASKAR, U. & MONTOGOMERY, W. (1978). Urinary lactic dehydrogenase isoenzyme IV and V in the differential diagnosis of cystitis and pyelonephritis. *J. Pediatr.*, **93**, 789.

ELHILANI, M.M., OLIVER, J.A., SHERWIN, A.L. & MACKINNON, K.J. (1967). Lactate dehydrogenase isoenzymes in hyperplasia and carcinoma of the prostate. A clinical study. *J. Urol.*, **98**, 686.

EVANS, M.J., EDDY, M. & PLUMMER, J. (1985). A comparative assessment of lactate dehydrogenase isoenzymes, $LDH_k$ and $LDH_5$. *J. Biol. Chem.*, **260**, 306.

FUJII, M., MORI, H., KATO, K. & TAKAHASHI, M. (1982). Cytochemical studies of LDH isoenzymes in experimental bladder tumors. *J. Urol.*, **128**, 1349.

FUJII, M., NISHIKAWA, A., TANAKA, T. & 7 others (1984). Cytochemical changes in lactate dehydrogenase isoenzymes in human brain tumours. *Acta Neurochirurgica*, **71**, 243.

FRIES, D., DELAVELLE, F., SIMONET, M., ECHARD, Y. & MATHIEU, D. (1977). Diagnostic topographique de l'infection urinaire par dosage de la fraction 5 de la lactico-deshydrogenase. *Nouv. Presse. Med.*, **6**, 3815.

GELDERMAN, A.H., GELBOIN, H.V. & PEACOCK, A.C. (1965). Lactic dehydrogenase isozymes in urine from patients with malignancies of the urinary bladder. *J. Lab. Clin. Med.*, **65**, 132.

GOLDMAN, R.D., KAPLAN, N.O. & HALL, T.C. (1964). Lactate dehydrogenase in human neoplastic tissues. *Cancer Res.*, **24**, 389.

IBSEN, K. & FISHMAN, W.H. (1979). Development gene expression in cancer. *Biochim. Biophys. Acta.*, **560**, 243.

LANGVAD, E. (1968). Lactate dehydrogenase isoenzyme patterns in bronchogenic carcinoma. *Eur. J. Cancer*, **4**, 170.

LORENTZ, W.B. & RESNICK, M. (1979). Comparison of urinary lactic dehydrogenase with antibody-coated bacteria in the urine sediment as means of localizing the site of urinary tract infection. *Pediatrics*, **64**, 672.

MACBETH, R.A.L. & BEKESI, J.G. (1962). Oxygen consumption and anaerobic glycolysis of human malignant and normal tissue. *Cancer Res.*, **22**, 224.

MALIK, G.M., CANAWATI, H.N., KEYSER, A.J., IBRAHIM, M.Z. & MONTOGOMERIE, J.Z. (1983). Correlation of urinary lactic dehydrogenase with polymorphonuclear leucocytes in urinary tract infections in patients with spinal cord injuries. *J. Infect. Dis.*, **147**, 161.

MATSUDA, M., OSAFUNE, M., NAKANO, E. & 10 others (1980). Lactate dehydrogenase in human renal carcinoma tissues. *Urol. Res.*, **8**, 201.

MIRABILE, C.S., BOWERS, G.N. & BERLIN, B.B. (1966). Urinary lactic dehydrogenase. A report based on 250 hospitalized patients. *J. Urol.*, **95**, 79.

MORIN, M.E. & HANCE, A.J. (1983). $LDH_k$, the lactate dehydrogenase associated with transformation by Kirsten sarcoma virus. A re-evaluation. *J. Biol. Chem.*, **258**, 2864.

MOTOMIYA, Y., YAMADA, K., MATSUSHIMA, S. & 6 others (1975). Studies on urinary isozymes of lactic dehydrogenase and $\beta$-glucuronidase in patients with bladder tumors. *Urol. Res.*, **3**, 41.

MOTOMIYA, Y., OHZONO, S., SHIOMI, T. & 6 others (1979). Studies on lactic dehydrogenase of patients with urinary bladder tumors. I. Urinary lactic dehydrogenase. *Invest. Urol.*, **17**, 120.

POSEY, L.E. & MORGAN, L.R. (1977). Urine enzyme activities in patients with transitional cell carcinoma of bladder. *Clin. Chim. Acta.*, **74**, 7.

ROSALKI, S.B. & WILKINSON, J.H. (1959). Urinary lactic dehydrogenase in renal diseases. *Lancet*, **ii**, 327.

TANAKA, T., FUJII, M., NISHIKAWA, A. & 12 others (1984). A cytochemical study of lactic dehydrogenase (LDH) isoenzymes in human lung cancer. *Cancer Detect. Prev.*, **7**, 65.

WACKER, W.E.C. & DORFMAN, L.E. (1962). Urinary lactic dehydrogenase. I. Screening method for detection of cancer of kidneys and bladder. *JAMA*, **181**, 972.